# Changes in Body Weight Percentile During Primary Anticancer Treatment in Children: A Retrospective Single-Center Study

**DOI:** 10.7759/cureus.117005

**Published:** 2026-09-27

**Authors:** Dorota Bartkowiak, Łukasz Kopoczynski, Michal Wiesner, Łukasz Wodzinski, Bartłomiej Jakubczyk, Adam Lasota, Kamil Boguszewski, Hubert Porazinski, Agnieszka Młynarska, Oskar Ratajski

**Affiliations:** 1 Medicine, Dr. Jan Biziel University Hospital No. 2, Bydgoszcz, POL; 2 Medicine, Polish Red Cross Maritime Hospital, Gdynia, POL; 3 Medicine, District Health Center, Malbork, POL; 4 Medicine, Florian Ceynowa Specialist Hospital, Wejherowo, POL; 5 Medicine, Antoni Jurasz University Hospital No. 1, Bydgoszcz, POL; 6 Cardiology, Pope John Paul II Independent Public Regional Hospital, Zamość, POL; 7 Medicine, Nowy Szpital in Nakło and Szubin, Bydgoszcz, POL; 8 Medicine, Dr. Jan Biziel University Hospital No. 2, Niemcz, POL; 9 Internal Medicine, Karol Marcinkowski University Hospital, Zielona Góra, POL

**Keywords:** body weight percentile, body weight trajectory, childhood cancer, growth charts, pediatric oncology

## Abstract

Objective

Body weight trajectories may change during anticancer treatment in children. This study aimed to evaluate changes in body weight percentile categories during primary anticancer treatment in pediatric oncology patients and to assess whether the magnitude and direction of these changes were associated with baseline body weight percentile category.

Materials and methods

This retrospective single-center study included 138 children diagnosed and treated for malignant disease between 2021 and 2025. Body weight was assessed at the initiation and at the completion of primary anticancer treatment using World Health Organization growth standards for children aged 0 to five years and Polish OLAF reference charts for children and adolescents aged six to 17 years. Each measurement was assigned to one of eight predefined centile channels, and continuous analyses were performed on the midpoints of these channels rather than on exact individual percentile values. Body weight percentile categories were analyzed, and changes between baseline and treatment completion were evaluated using the Wilcoxon signed-rank test and the Kruskal-Wallis test.

Results

Complete paired anthropometric data were available for 138 patients. The mean body weight percentile (channel midpoint) decreased from 56.1 at treatment initiation to 54.65 at treatment completion, while the median remained 62.5 at both assessments. The difference was not statistically significant (p = 0.5254). The proportion of children below the 10th percentile increased from 10.14% to 13.04%, whereas the proportion above the 90th percentile decreased from 22.46% to 16.67%. Most patients remained within the same percentile category or changed by only one category. Baseline body weight percentile category was significantly associated with the distribution of category-change scores (H = 15.25, p = 0.033, η² = 0.06). Children with lower baseline percentiles tended to shift upward, whereas those with higher baseline percentiles tended to shift downward; this pattern should be interpreted with caution, as it may partly reflect regression to the mean and the structurally restricted range of possible change in the extreme categories, in which upward or downward shifts are arithmetically limited.

Conclusions

Body weight percentiles showed no statistically significant overall change at the population level during primary anticancer treatment; however, individual changes in percentile categories were common. Regular assessment of body weight percentile trajectories may identify changes that are not apparent from group-level measures and may prompt further nutritional assessment. Body weight percentile alone does not provide a comprehensive assessment of nutritional status.

## Introduction

Childhood cancers are a highly heterogeneous group of diseases in terms of histological type and anatomical location. The spectrum and distribution of cancer types in children differ considerably from those observed in adults [1]. The diagnostic pathway for childhood cancer may involve multiple healthcare contacts and variable intervals between first presentation and diagnosis [2]. Childhood cancer is managed with disease-specific oncological therapy tailored to the diagnosis, together with supportive care for treatment-related complications [3].

Growth monitoring based on age- and sex-specific reference charts is an integral part of pediatric assessment. Percentile curves allow the anthropometric measurements of a child to be compared with those of a reference population and permit the child’s developmental trajectory to be monitored over time. Remaining within the same percentile channel generally indicates a relatively stable growth pattern, whereas crossing percentile channels may indicate a change that warrants further clinical evaluation.

The interpretation of pediatric anthropometric measurements requires age-appropriate and population-relevant reference standards. The World Health Organization growth standards provide internationally recognized reference values for young children, whereas the OLAF project provides updated, nationally representative references for Polish children and adolescents [4,5]. The use of these complementary standards permits anthropometric assessment across the wide age range encountered in pediatric oncology. This study aimed to evaluate changes in body weight percentile categories during primary anticancer treatment in children and to assess whether the magnitude and direction of these changes were associated with baseline body weight percentile category.

## Materials and methods

A retrospective study was conducted on children in the Department of Pediatrics, Hematology and Oncology at Dr. Antoni Jurasz University Hospital No. 1, Bydgoszcz, Poland, who were diagnosed and treated for cancer between 2021 and 2025. A total of 161 patients were assessed for eligibility. Twenty-two patients were excluded: three because the final diagnosis was a benign lesion rather than a malignant neoplasm, 16 because of incomplete medical documentation, and three because of death before completion of primary treatment. The final study cohort comprised 139 patients. One of these patients had not completed primary anticancer treatment at the time of data extraction; consequently, neither the duration of primary treatment nor a body weight measurement at completion of treatment was available for this patient, and the patient was excluded from all analyses. All analyses reported below are therefore based on 138 patients, and all percentages were calculated using this denominator.

The study was based on medical records of patients treated according to the current therapeutic protocol. For the purposes of this study, primary anticancer treatment was defined as the initial disease-directed treatment administered according to the protocol applicable to the patient's diagnosis. Completion of primary treatment was defined as completion of the planned initial treatment phase according to the disease-specific therapeutic protocol. Baseline body weight was defined as the measurement recorded at the initiation of primary anticancer treatment, whereas the final body weight was defined as the measurement recorded at completion of the primary treatment phase. Body weight measurements were extracted retrospectively from the patients’ medical records. For each patient, the measurement recorded at the initiation of primary treatment and the measurement recorded at completion of primary treatment were selected for analysis. Body weight measurements were performed as part of routine clinical care using medical weighing equipment appropriate for the patient’s age. Body weight was converted to age- and sex-specific percentile values using the reference charts applicable to the child’s age. The study was carried out in accordance with the Declaration of Helsinki and approved by the local Bioethics Committee.

A total of 139 children were assessed for inclusion. After exclusion of one boy who had not completed primary anticancer treatment, 138 children were analyzed, including 70 boys (50.72%) and 68 girls (49.28%). The mean age at diagnosis was 8.0 ± 5.6 years, and the median age was 6.0 years (range 0-17 years). Information on the duration of primary treatment was available for 138 patients. The mean duration of treatment was 7.3 months, with a median of 7.2 months (range 0.0-32.5 months). Treatment durations shorter than one month were recorded as 0.0 months after rounding; therefore, a value of 0.0 months did not indicate absence of treatment. Baseline body weight assessment was available for 138 patients; the mean body weight percentile was 56.1, and the median was 62.5.The wide range of observed body weight percentile values (1.5th-98.5th percentile) demonstrated substantial variability in baseline body weight percentiles within the study population. Most patients had body weight between the 10th and 90th percentiles, while some had values at the lower or upper extremes of the percentile distribution. Detailed characteristics of the study population are presented in Tables 1, 2. Inclusion and exclusion criteria are presented in Table 3.

**Table 1:** Characteristics of the study population

Variables	N	M	Me	Min	Max
Age (years)	138	8.0	6.0	0.0	17.0
Duration of primary treatment (months)	138	7.3	7.2	0.0	32.5
Baseline body weight (percentile, channel midpoint)	138	56.1	62.5	1.5	98.5

**Table 2:** Sex distribution of the study group

Factor	Category	Number of patients	Percentage of Group (%)
Sex	Male	70	50.72
Sex	Female	68	49.28

**Table 3:** Inclusion and exclusion criteria A total of 161 patients were assessed for eligibility. Twenty-two patients were excluded: three because the final diagnosis was a benign lesion rather than a malignant neoplasm, 16 because of incomplete medical documentation, and three because of death before completion of primary treatment. The final study cohort comprised 139 patients, of whom 138 were analyzed; one patient was excluded because primary anticancer treatment had not been completed at the time of data extraction.

Criterion	Inclusion	Exclusion
Age	0-17 years	≥18 years
Diagnosis	Diagnosed malignant neoplasm	Benign lesion rather than malignant neoplasm (n = 3)
Study period	Diagnosis and treatment between 2021 and 2025	Outside the study period
Medical documentation	Sufficient medical documentation for the study analysis	Incomplete medical documentation (n = 16)
Treatment completion	Completion of primary anticancer treatment	Death before completion of primary treatment (n = 3)

A dual methodological approach was used to assess the body weight of patients: WHO growth standards were applied to children aged 0 to five years, whereas Polish OLAF reference charts were applied to children aged six to 17 years [4,5]. The choice of the OLAF standards was based on their greater suitability for the Polish pediatric population, as illustrated in Table 4. Percentile channels were defined according to the principal centile boundaries used in the selected body weight references: <3rd, 3rd-10th, 10th-25th, 25th-50th, 50th-75th, 75th-90th, 90th-97th and >97th percentiles. We assessed body weight percentile categories at the beginning and after anticancer treatment. 

**Table 4:** Comparison of anthropometric standards used in the study

Characteristic	WHO standards (2006/2007) [4]	Polish OLAF standards (2010) [5]
Age group	0-19 years ( in the study: 0-5 years)	6-19 years (in the study: 6-17 years)
Reference population	International (6 countries)	Poland (nationwide study)
Sample size	~8,500 children	>17,000 children
Advantages	Universal standard, high sensitivity <5years of age	Accounts for the local secular trend
Application in the study	Children aged 0–5 years	Children and adolescents aged 6–17 years

Percentile categories were used as an ordinal descriptive measure to characterize individual changes in body weight position on age- and sex-specific growth charts during primary anticancer treatment. This approach was selected to facilitate the clinical description of individual body weight trajectories and category transitions. The analysis of category transitions was not intended to represent equal absolute or standardized changes in body weight. It should be emphasized that no fully continuous percentile variable was analyzed: for the numerical summaries and the paired comparison, each observation was represented by the midpoint of its centile channel (1.5 for the below-3rd, 6.5 for the 3rd-10th, 17.5 for the 10th-25th, 37.5 for the 25th-50th, 62.5 for the 50th-75th, 82.5 for the 75th-90th, 93.5 for the 90th-97th and 98.5 for the above-97th percentile channel). Consequently, all body weight percentile values reported in this article, including the minimum of 1.5 and the maximum of 98.5, represent channel midpoints rather than exact individual percentile values. The paired comparison and category-transition analysis are based on the same discretized data and are therefore not independent of one another.

Both children and parents were provided with dietary advice on high-protein, easy-to-digest foods during treatment and hospitalization. If gastrointestinal dysfunction was observed, a dairy-free low-fiber diet was introduced. In some patients, dietary changes were required because of pancreatic, hepatic, or renal irritation or renal dysfunction. When the hospital diet was insufficient for the child’s nutritional needs, oral nutritional supplements were provided. When oral intake was less than 50% of the estimated nutritional needs, enteral nutrition (if possible) or partial or full parenteral nutrition was initiated. Exposure to specific nutritional interventions was not recorded systematically and was not analyzed as a study variable.

The data were analyzed statistically. All continuous variables were summarized by mean (M), standard deviation (SD), median (Me), and range (minimum to maximum). Categorical variables were reported as frequencies (n) and percentages (%). Unless otherwise specified, percentages were calculated using the analyzed cohort as the denominator. For descriptive statistics of the change score Δ within baseline categories, M denotes the mean, SD the standard deviation, R the range (maximum minus minimum), Q25 and Q75 the first and third quartiles, respectively, and Me the median. The distribution of the baseline body weight percentiles was described numerically and graphically. The change in body weight percentile category between baseline and completion of primary treatment was expressed as the number of percentile categories crossed (Δ), calculated as the final category rank minus the baseline category rank. Positive values indicated an upward shift, negative values indicated a downward shift, and a value of zero indicated no change in category. Because percentile data were ordinal and normality could not be assumed, the Wilcoxon signed-rank test for paired samples was used to assess changes in body weight percentile between the beginning and the end of primary treatment. Only patients with complete anthropometric data at both time points were included in the paired analysis. The Wilcoxon test statistic (Z) and the corresponding p-value were reported. We also investigated changes across three descriptive body weight percentile groups: <10th percentile, 10th-90th percentile, and >90th percentile. These groups were used for descriptive purposes and were not interpreted as diagnostic categories of undernutrition, overweight, or obesity. The association between baseline body weight percentile category and the magnitude and direction of change in percentile category was assessed using the Kruskal-Wallis test. The test statistic (H) and corresponding p-value were reported, with eta squared (η²) used as a measure of effect size. Because the possible range of Δ is bounded by the position of the baseline category (children in the lowest category cannot shift downward and those in the highest category cannot shift upward), this comparison was regarded as descriptive and hypothesis-generating rather than confirmatory. A p-value <0.05 was considered statistically significant. No adjustment for multiple comparisons and no sample size or power calculation were performed. Statistical analyses were performed using Statistica software, version 13.3 (TIBCO Software Inc., Palo Alto, CA, USA).

## Results

Characteristics of the study population

Regarding cancer type, 42 of 138 patients (30.43%) were diagnosed with solid tumors and 96 (69.57%) with hematological malignancies. Chemotherapy was administered to 131 patients (94.93%), 35 patients (25.36%) underwent surgery, 14 (10.14%) underwent hematopoietic stem cell transplantation (HSCT), and 15 (10.87%) received radiotherapy. Ten patients (7.25%) required treatment in the intensive care unit (ICU). Treatment modalities were not mutually exclusive, as individual patients could receive more than one treatment modality during primary anticancer treatment. The present analysis was not designed to compare body weight percentile trajectories between diagnostic groups or treatment modalities.

Baseline body weight percentile distribution

At baseline, 93 patients (67.39%) had body weight between the 10th and 90th percentiles. The largest subgroups were children in the 25th-50th percentile category (34 patients, 24.64%), followed by the 50th-75th percentile category (29 patients, 21.01%) and the 75th-90th percentile category (17 patients, 12.32%). Thirteen patients (9.42%) were in the 10th-25th percentile category. Fourteen children (10.14%) had body weight below the 10th percentile, including six children (4.35%) below the 3rd percentile and eight children (5.80%) between the 3rd and 10th percentiles. Thirty-one patients (22.46%) had body weight above the 90th percentile, including 19 children (13.77%) between the 90th and 97th percentiles and 12 children (8.70%) above the 97th percentile. Complete baseline and final body weight data were available for all 138 analyzed patients. Thus, most patients had baseline body weight between the 10th and 90th percentiles, whereas approximately one-third had values outside this range. The distribution of baseline body weight percentile categories is shown in Table 5.

**Table 5:** Distribution of baseline body weight percentiles Rows indicate the centile channel occupied at the initiation of primary anticancer treatment. Percentages were calculated using the analyzed cohort as the denominator (n = 138).

Baseline body weight percentile channels	Number of patients	Percent (%)
<3	6	4.35
3-10	8	5.80
10-25	13	9.42
25-50	34	24.64
50-75	29	21.01
75-90	17	12.32
90-97	19	13.77
>97	12	8.70

Changes in body weight during primary cancer treatment

A total of 138 patients with complete anthropometric data at both assessment time points were included in the study. The mean body weight percentile was 56.1 when starting primary treatment, and 54.65 when completing primary treatment. The median body weight percentile was 62.5 in both assessments, and the range of body weight percentiles was the same (1.5th to 98.5th percentile). As stated in the Methods, these values are midpoints of the corresponding centile channels. Compared with the baseline and primary treatment data, the Wilcoxon signed-rank test showed no difference in body weight percentile between the two analyses (Z = 0.63, p = 0.5254). Although there was a small decrease in the mean body weight percentile, this was not statistically significant. The absence of a statistically significant change in the median body weight percentile should not be interpreted as evidence that all patients maintained stable body weight percentile positions. Growth charts are designed not only to classify a child at a single time point but also to evaluate the trajectory of repeated measurements. Consequently, movement across percentile channels may reveal clinically relevant individual changes that remain obscured when only group-level central tendency measures are considered [4,5]. The changes in body weight percentile between the beginning and the end of primary treatment are shown in Table 6 and Figure 1.

**Table 6:** The changes in body weight percentile between the beginning and the end of primary treatment Wilcoxon signed-rank test for paired samples was used to compare body weight percentiles at baseline and after primary treatment. Z = test statistic; p = p-value.

	N	M	Me	Min	Max	Z	p
Baseline body weight (percentile)	138	56.1	62.5	1.5	98.5	0.63	0.5254
Body weight after treatment (percentile)	138	54.65	62.5	1.5	98.5		

**Figure 1:**
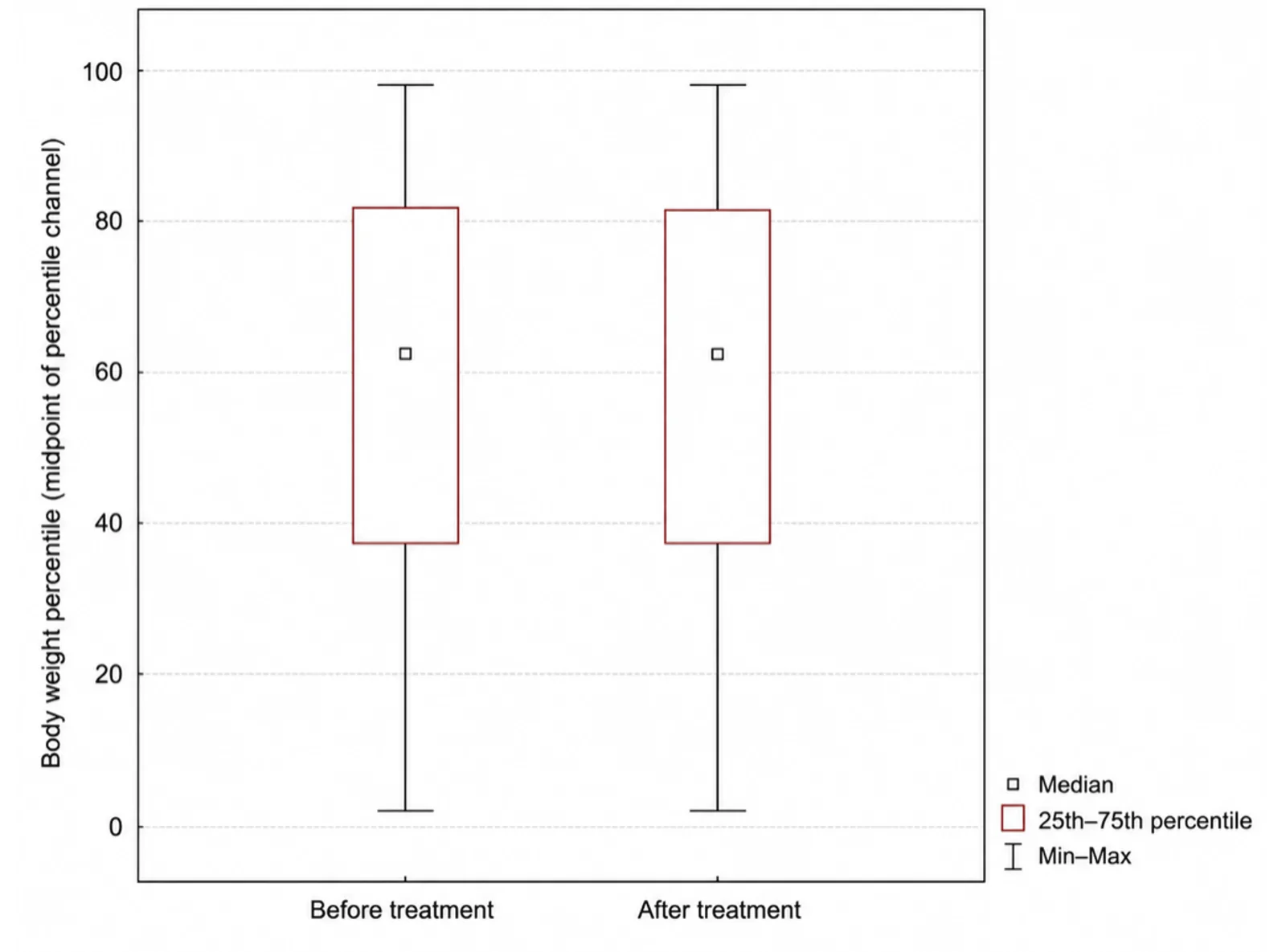
Distribution of body weight percentiles at baseline and at completion of primary anticancer treatment test Wilcoxon ; Z = 0.63; p = 0.525 Values plotted are midpoints of the predefined centile channels (n = 138).

Change in the distribution of body weight percentiles during primary treatment

We included baseline body weight data for 138 children and follow-up data for 138 children. The detailed distribution of body weight percentile categories before and after treatment is presented in Table 7. Before treatment, the majority of patients were in the 25th to 50th percentile (24.64%). At the end of treatment, the largest subgroup in the analyzed group consisted of children with body weight between the 50th and 75th percentiles (25.36%).. At both time points, most patients were in the 10th-90th percentile range for age and sex. There was a slight increase in the number of children in the lowest percentile categories after treatment. The percentage of patients below the 3rd percentile increased from 4.35% to 5.07%. The percentage in the 3rd-10th percentile increased from 5.80% to 7.97%. The proportion of children in the 10th-25th percentile decreased from 9.42% to 7.97%. The proportion in the 25th-50th percentile decreased from 24.64% to 21.74%. On the other hand, the percentage of children in the 50th-75th percentile category climbed from 21.01% to 25.36%, while the percentage in the 75th-90th percentile category rose from 12.32% to 15.22%. The percentage of children with body weight above the 90th percentile decreased in the 90th-97th percentile range (13.77% to 8.70%) and in the >97th percentile range (8.70% to 7.97%). These results indicate a small decrease in the proportion of patients with body weight above the 90th percentile during primary cancer treatment. As shown in Table 8, 67.39% of patients had body weight between the 10th and 90th percentiles before treatment, increasing slightly to 70.29% after treatment. The proportion of children with body weight below the 10th percentile increased from 10.14% to 13.04%, whereas the proportion with body weight above the 90th percentile decreased from 22.46% to 16.67%. Overall, most patients had body weight between the 10th and 90th percentiles at both assessment points. These are descriptive proportions; no statistical comparison of the two distributions was performed.

**Table 7:** The distribution of body weight percentiles before and after treatment Note: Percentages were calculated using the analyzed cohort as the denominator (n = 138).

	Before treatment	Before treatment	After treatment	After treatment
Percentiles	Number of patients	Group percentage (%)	Number of patients	Group percentage (%)
<3	6	4.35	7	5.07
3-10	8	5.80	11	7.97
10-25	13	9.42	11	7.97
25-50	34	24.64	30	21.74
50-75	29	21.01	35	25.36
75-90	17	12.32	21	15.22
90-97	19	13.77	12	8.70
>97	12	8.70	11	7.97

**Table 8:** Distribution of patients according to body weight percentile groups Note: These groups are descriptive body weight percentile groups and were not interpreted as diagnostic categories of undernutrition, overweight or obesity. Percentages were calculated using the analyzed cohort as the denominator (n = 138).

Assessment	Before treatment	Before treatment	After treatment	After treatment
	Number of Patients	Percentage of Group (%)	Number of Patients	Percentage of Group (%)
<10th percentile	14	10.14	18	13.04
10th–90th percentile	93	67.39	97	70.29
>90th percentile	31	22.46	23	16.67

Pattern of changes in body weight percentile categories

The pattern of changes in body weight percentile categories during primary cancer treatment is presented in Table 9. The largest subgroup consisted of patients whose body weight remained within the same percentile category throughout treatment (64/138, 46.38%). A decline of one percentile category was observed in 29 children (21.01%), whereas an increase of one percentile category occurred in 25 children (18.12%). Changes involving two percentile categories were considerably less frequent, observed in 11 children (7.97%) in the direction of a downward shift and in 7 children (5.07%) in the direction of an upward shift. Shifts of three or four percentile categories were rare and together accounted for less than 2% of the study population. These findings indicate that body weight remained stable or changed only slightly in most patients during primary cancer treatment. To determine whether the magnitude of change depended on the baseline body weight percentile category, a Kruskal-Wallis analysis was performed (Table 10). A statistically significant association was found between baseline body weight percentile category and the distribution of category-change scores (Δ) (H = 15.25, p = 0.033, η² = 0.06). This test compares Δ across baseline categories; it does not indicate that the individual category transitions were themselves statistically significant, and the overall paired comparison of body weight percentile was not significant. Patients who initiated treatment with body weight below the 50th percentile showed a slight tendency toward an upward shift in percentile category. The greatest mean increase was observed among children with body weight below the 3rd percentile (Δ = 0.33) and those within the 3rd-10th percentile range (Δ = 0.50). In contrast, patients whose baseline body weight exceeded the 50th percentile demonstrated a tendency toward downward shifts in percentile category, with the most pronounced decrease observed in the 90th-97th percentile group (Δ = -0.68). Despite these differences, the median change remained close to zero across all groups analyzed, indicating that most children did not change their body weight percentile category during treatment. These subgroup estimates are based on small numbers of patients (n = 6 to 34 per category) and should be regarded as imprecise.

**Table 9:** Changes in body weight percentile channels

Size of changes	Number of patients	Group percentage (%)
-4	1	0.72
-3	1	0.72
-2	11	7.97
-1	29	21.01
0	64	46.38
1	25	18.12
2	7	5.07

**Table 10:** Changes in body weight percentile category (Δ) according to baseline body weight percentile category NOTE: Δ = number of body weight percentile categories crossed between baseline and completion of primary treatment, calculated as the final category rank minus the baseline category rank; positive values indicate an upward shift and negative values indicate a downward shift; H = Kruskal–Wallis test statistic; η²H = effect size measure.

Body weight percentile	M	n	SD	Min	Max	R	Q₂₅	Me	Q₇₅	H	p	η²H
<3	0.333	6	0.516	0.00	1.000	1	0.00	0.00	1.000	15.25	0.0330	0.06
3–10	0.500	8	1.195	−1.00	2.000	3	−0.50	0.50	1.500			
10–25	0.000	13	1.080	−2.00	2.000	4	−1.00	0.00	1.000			
25–50	0.118	34	0.977	−2.00	2.000	4	0.00	0.00	1.000			
50–75	−0.036	29	0.922	−3.00	2.000	5	0.00	0.00	0.000			
75–90	−0.412	17	1.004	−2.00	2.000	4	−1.00	0.00	0.000			
90–97	−0.684	19	1.250	−4.00	1.000	5	−1.00	−1.00	0.000			
>97	−0.500	12	0.674	−2.00	0.000	2	−1.00	0.00	0.000			
Overall	−0.124	138	1.032	−4.00	2.000	6	−1.00	0.00	0.000			

Figure 2 illustrates the distribution of changes across individual body weight percentile categories. The greatest variability was observed among patients with extreme baseline values, namely those below the 10th percentile and above the 90th percentile. In these groups, the range of observed changes was considerably wider than in children with baseline body weight within 10th-90th percentile. In contrast, patients who initiated treatment with body weight between the 10th and 90th percentiles exhibited only minor changes, with most values clustering around zero, indicating relative stability of body weight percentile category throughout primary cancer treatment.

**Figure 2:**
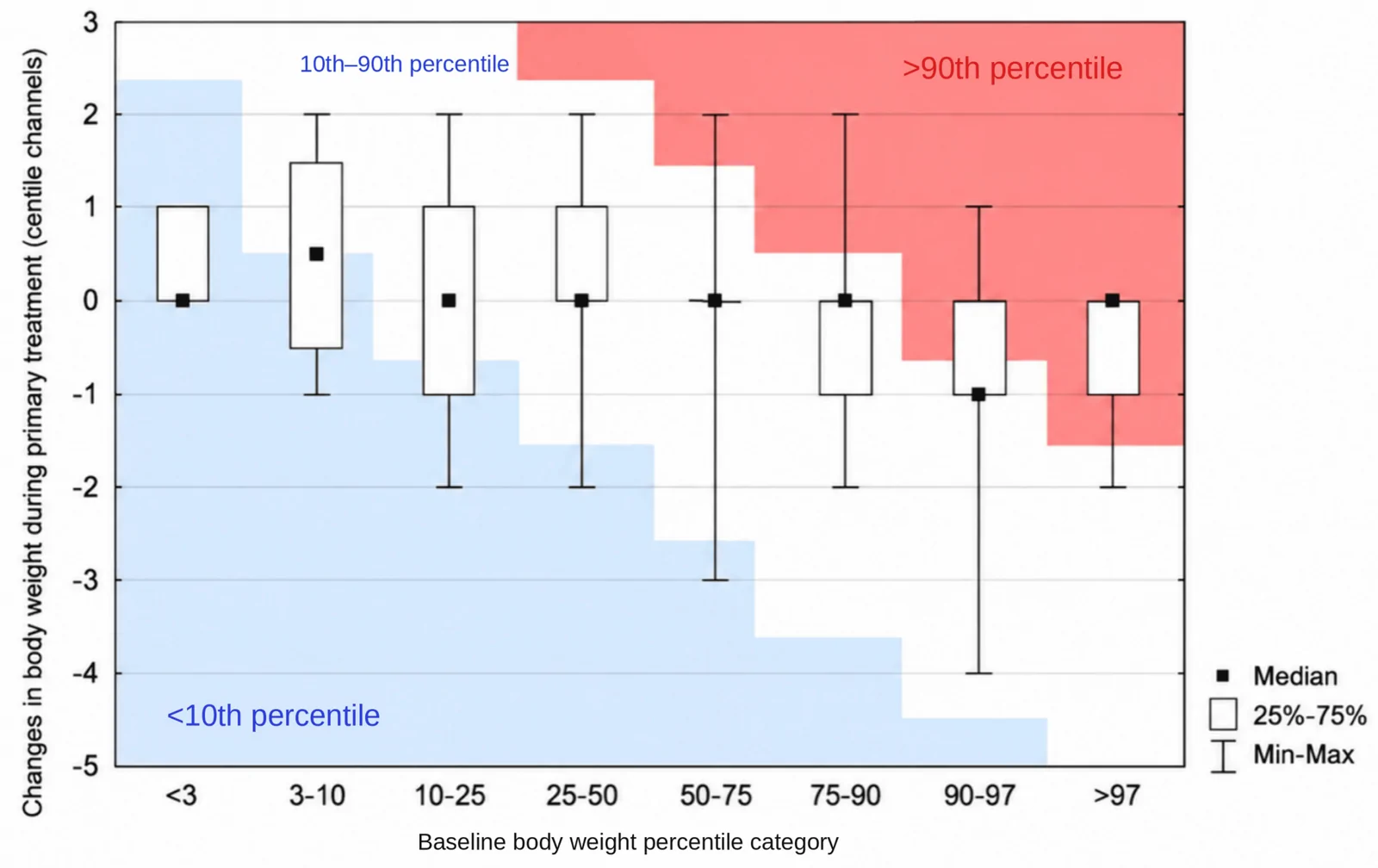
Changes across individual body weight percentile categories Note: The white area indicates the 10th–90th percentile range, the red area indicates values >90th percentile, and the blue area indicates values <10th percentile.

The wider range of changes observed among children with extreme baseline percentiles may partly reflect both greater clinical vulnerability and the statistical tendency of extreme measurements to move toward more central values during follow-up. In addition, the possible values of Δ are structurally constrained by the baseline category, so a difference in Δ between baseline categories is expected on arithmetical grounds alone. Nevertheless, these findings remain clinically important because children who begin treatment at the lower end of the weight distribution have limited nutritional reserves, whereas those with body weight at the upper extreme of the distribution may be at increased risk of treatment-related metabolic complications. Therefore, particular attention should be paid to patients presenting with baseline anthropometric values outside the 10th-90th percentile range.

## Discussion

The demographics of the population studied in the context of the epidemiology of childhood cancer

Childhood cancer is a complex disease, and its histopathological characteristics are different - for example, the age of diagnosis of cancer, location, and course of clinical development. Childhood cancer differs from adult cancer, as most childhood malignancies arise from embryonal tissues and from the hematopoietic system, and they differ in age at diagnosis, anatomical location and clinical course. The distribution of malignancies in our population was similar to that reported in global epidemiological data from the International Incidence of Childhood Cancer, Volume III (IICC-3), published by the International Agency for Research on Cancer (IARC) [6]. Leukemia was the most common childhood cancer, followed by CNS tumors and lymphomas. The remaining malignancies were heterogeneous and belonged to ICCC-3 (International Classification of Childhood Cancer, Third Edition). In the group we identified, hematological malignancies accounted for 70% of all diagnoses and solid tumors for 30%. The study was carried out in a single tertiary pediatric oncology center, but the trend is consistent with the global prevalence of hematological malignancies in children. In 2022, there were 211,080 new cases of childhood cancer and 78,441 related deaths worldwide. Acute Lymphoblastic Leukemia was the most commonly diagnosed malignancy, accounting for 66.73% of all leukemia cases and an age-standardized incidence rate (ASR) of 2.04 per 100,000 population [7].

The mean age at diagnosis was 8.0 ± 5.6 years, and the median age was 6 years, which is consistent with the age distribution typically observed in pediatric oncology populations. This is higher than the peak incidence age of acute lymphoblastic leukemia, between two and five years of age [8].

The mean duration of primary treatment in our group was 7.3 months, which is the intensive treatment period in most modern pediatric oncology protocols. The treatment of acute lymphoblastic leukemia is characterized by long induction, consolidation, and re-induction phases. The treatment of solid tumors depends on the type of cancer and is commonly combined with surgery and/or radiotherapy. Despite improvements in the treatment options and the application of non-toxic drugs, chemotherapy and hematopoietic stem cell transplantation (HSCT) are the main methods of treatment for ALL. New clinical treatments have been discovered and implemented for treatment with significant effects on overall survival (OS) [9]. No matter the diagnosis, the first months of treatment are associated with the most severe treatment-related adverse effects that may compromise nutritional status. Nutritional status is also a key determinant of both prognosis and survival of pediatric oncology patients. Nutritional screening, comprehensive nutritional assessment, and regular monitoring should be done frequently to minimize the adverse effects of malnutrition on health outcomes [10]. Nutritional monitoring is a crucial component of pediatric oncology care, as undernutrition and excessive weight may affect chemotherapy tolerance, and chemotherapy tolerance is associated with cancer patients having a greater risk of developing infectious complications, longer hospitalization, and poorer quality of life. Therefore, every child diagnosed with cancer should be evaluated and should be given nutritional assistance throughout treatment [11].

In this study, most of the children started their treatment with body weight between the 10th and 90th percentiles for age and sex. However, about one-third of patients already had body weight percentile values outside this range at the time of diagnosis. This indicates that deviations in body weight percentile are present before the start of anticancer therapy and are not solely a consequence of chemotherapy. A high prevalence of malnutrition at diagnosis has also been reported in previous studies [12-14], although those studies applied more comprehensive nutritional assessment methods than body weight-for-age percentile alone, and the present data do not allow a diagnosis of malnutrition to be made.

Overall, the characteristics of the population in this study suggest that our sample is representative of children being treated for malignant diseases at tertiary referral centers in the current epidemiological context of childhood cancer. These findings lay a base for further studies of nutritional changes that occur during treatment and what factors might drive them to their effect and magnitude.

The importance of nutritional assessment using growth charts

In children, nutritional status assessment is crucial to understand how growth and development can vary and develop. While in adults the body mass index (BMI) and absolute values of the body are mainly used, pediatric nutritional assessment is based on growth charts, and anthropometric measurements can be interpreted according to the child’s age and sex. The concept of measuring growth using percentile distributions has been around for over a century. The first growth charts were developed in the early twentieth century, but the modern growth charts evolved after the large population-based studies in Europe and North America. A major milestone was the publication of the World Health Organization (WHO) Child Growth Standards in 2006, based on the Multicentre Growth Reference Study, which established international growth standards for children up to five years old. In subsequent years, many European countries developed their own national growth charts reflecting the characteristics of their local populations.

In Poland, growth charts developed by the Institute of Mother and Child, as well as by research groups from Warsaw and Kraków, were used for many years. In the present study, WHO growth standards were applied to children aged 0-5 years, whereas Polish OLAF reference charts were applied to children aged 6-17 years [4,5]. This approach allows for a more accurate assessment of physical development in relation to the reference population and is recommended in routine pediatric practice. International growth charts for children older than 5 years have limitations, as study designs and populations have not been the same, which has prevented a universal standard for nutritional status from being established in the international field. Moreover, it is not easy to create international growth charts that are appropriate for children and adolescents to keep up through the growth period. So that they do not take into account the differences in genetic, cultural, socioeconomic, and body composition among children and adolescents from different ethnic groups [15]. This approach was used to assess changes in body weight while accounting for physiological growth and normal age and sex differences, which would not be possible using absolute body weight.

What is striking about this study is that most of the children were still between the 10th and 90th percentiles at diagnosis and at the end of primary treatment. This indicates that body weight remained between the 10th and 90th percentiles in a substantial proportion of patients at both assessment time points. At the same time, the analysis demonstrated shifts between individual body weight percentile categories, indicating that some children experienced upward or downward changes in their body weight percentile trajectories. The absence of a statistically significant group-level difference is compatible with the occurrence of individual upward and downward category shifts that may offset one another when only central tendency measures are examined. The category-transition analysis provides complementary descriptive information; it does not constitute an additional statistical test demonstrating that individual transitions were significant. These results highlight that mean percentile values alone may not adequately capture individual changes during treatment, and that assessment of individual body weight percentile trajectories provides complementary information that may be obscured when only group-level measures are considered.

Our findings further support the usefulness of growth chart assessment as a tool for monitoring children undergoing cancer treatment. Regular evaluation of a child’s position on growth charts may facilitate the early identification of potentially clinically relevant changes in body weight trajectory, prompting further nutritional assessment when indicated.

Changes in body weight percentile during anticancer treatment

One of the most important findings of the present study is that, despite intensive anticancer therapy, there was no statistically significant change in the median body weight percentile between the initiation and completion of primary treatment. This finding indicates that there was no statistically significant overall change in body weight percentile at the population level. However, individual patient trajectories showed changes in body weight percentile categories, and the distribution of category-change scores differed according to baseline body weight percentile category. Thus, although the mean body weight percentile for the whole study population was relatively stable, many children changed their body weight percentile category in both directions.

This phenomenon reflects the marked heterogeneity of pediatric oncology patients. Nutrition changes are influenced not only by malignancy type and treatment protocol, tumor location, chemotherapy intensity, glucocorticoid therapy, surgery, and radiation, but also by treatment-related symptoms including gastrointestinal mucositis, nausea, vomiting, and infections. Children with cancer are particularly vulnerable to malnutrition, a condition that is caused by poor energy balance due to the interaction between the underlying disease and treatment. Malnutrition is associated with worse treatment outcomes and poorer quality of life [16]. In contrast, some children experience excessive weight gain because of lack of physical activity, glucocorticoid-induced hyperphagia, and metabolic disorders. These mechanisms are derived from the literature; the present study did not analyze the association between individual treatment components and body weight percentile trajectories.

The present findings demonstrate that analysis based solely on mean anthropometric values may fail to capture the true extent of changes in body weight percentile position occurring during treatment. Opposing trends observed in individual patients may offset one another at the population level, resulting in no statistically significant differences in mean values. Therefore, individual body weight percentile trajectories may provide complementary information that can be obscured when only group-level measures are considered. However, a comprehensive nutritional assessment should include anthropometric, biochemical, clinical, and dietary parameters. Furthermore, regular nutritional monitoring should be maintained throughout treatment and survivorship to facilitate timely implementation and evaluation of nutritional interventions [17].

The results of our study are consistent with those of G. Özalp Gerçeker [18], who demonstrated that the nutritional status of children with cancer during long-term hospitalization is very diverse and depends on many factors. Increasingly, nutritional care is regarded not only as a means of preventing malnutrition but also to promote normal growth and development and to minimize undernutrition and excessive weight gain.

After primary treatment, most children had body weight between the 10th and 90th percentiles. These findings were observed in the context of the nutritional monitoring and supportive-care practices used at our center, including dietary counseling and enteral or parenteral nutritional support when clinically indicated. Because exposure to specific nutritional interventions was not recorded as a study variable and no comparator group was available, no conclusion about the effectiveness of these practices can be drawn. At the same time, the observed individual changes in body weight percentile categories indicate that changes in body weight percentile position may occur even among patients whose body weight remained between the 10th and 90th percentiles, supporting the need for regular monitoring and further nutritional assessment when indicated.

The role of the multidisciplinary team in pediatric oncology has become increasingly important. The multidisciplinary care of physicians, nurses, clinical dietitians, when appropriate, psychologists, and physiotherapists helps in identifying nutritional abnormalities early and providing effective treatment by changing diet, providing oral nutrition, enteral nutrition, or parenteral nutrition. Multidisciplinary nutritional care may support the identification and management of nutritional problems during anticancer treatment. Depending on individual clinical needs, nutritional management may include dietary modification, oral nutritional supplements, enteral nutrition, or parenteral nutrition.

These findings emphasize the importance of systematic anthropometric monitoring throughout the entire course of anticancer therapy. A single anthropometric assessment, performed either at diagnosis or after treatment completion, may not adequately reflect the dynamic nutritional changes that occur during therapy. Regular monitoring of a child’s position on growth charts enables the early detection of unfavorable trends. Regular monitoring of body weight percentile trajectories may facilitate the early identification of unfavorable changes and help identify patients who require more comprehensive nutritional assessment and individualized nutritional support.

Limitations

This study has several limitations. First, its retrospective, single-center design limits the generalizability of the findings. In addition, the requirement for sufficient medical documentation and completion of primary treatment may have introduced selection and survivorship bias. Of the 161 patients assessed for eligibility, 22 were excluded, including 16 because of incomplete medical documentation and three because of death before completion of primary treatment; three additional patients were excluded because the final diagnosis was a benign lesion rather than a malignant neoplasm. In particular, the exclusion of patients who died before completion of primary treatment may have resulted in underrepresentation of patients with the most severe disease course. In addition, one further patient was excluded after cohort assembly because primary anticancer treatment had not been completed at the time of data extraction, so that 138 of the 139 patients in the study cohort were analyzed.

Second, the study population was heterogeneous with respect to cancer diagnosis, treatment duration, and treatment modalities, and the present analysis was not designed to determine the independent effects of specific diagnoses or treatments on body weight trajectories. Although data on cancer type and treatment modalities were available, no subgroup or multivariable analysis was performed; comparisons between hematological malignancies and solid tumors, between corticosteroid-containing and non-steroid regimens, and according to treatment duration should be addressed in future studies. 

Third, the assessment was based primarily on body weight-for-age percentiles. Weight percentile alone does not account for height, body proportions, body composition, or growth velocity and therefore should not be interpreted as a comprehensive measure of nutritional status. BMI-for-age, weight-for-height, height-for-age, mid-upper arm circumference, and body-composition measures were not included in the present analysis. In addition, percentile categories are ordinal and nonlinear; therefore, crossing one percentile category does not necessarily represent the same absolute or standardized change across different parts of the weight distribution. Age- and sex-standardized z-scores would provide a more uniform continuous measure of longitudinal change and should be considered in future studies. In addition, because body weight was measured as part of routine clinical care and recorded retrospectively, the type and calibration of the weighing equipment and the exact measurement conditions were not uniformly documented.

Fourth, body weight percentile values were analyzed as midpoints of the predefined centile channels rather than as exact individual percentile values. The mean and median values reported in Tables 1 and 6, the range of 1.5 to 98.5, and the distributions shown in Figure 1 therefore reflect discretized data. As a consequence, the paired Wilcoxon comparison and the category-transition analysis were performed on the same underlying variable and do not represent independent continuous and categorical analyses; the paired test also has reduced sensitivity to within-channel changes in body weight.

Fifth, the possible values of the change score Δ are bounded by the baseline category: children in the lowest category cannot shift downward, and those in the highest category cannot shift upward. A difference in Δ between baseline categories is therefore expected on arithmetical grounds, and the Kruskal-Wallis result should not be interpreted as evidence of a biological gradient.

Sixth, several baseline categories contained small numbers of patients (n = 6 to 19 in the extreme categories) and no sample size or power calculation was performed, so subgroup estimates are imprecise.

Finally, the observed tendency of patients with extreme baseline percentiles to move toward more central categories may partly reflect regression to the mean and should therefore be interpreted cautiously.

## Conclusions

Body weight percentiles showed no statistically significant overall change at the population level during primary anticancer treatment; however, individual patients exhibited changes in body weight percentile categories. The distribution of category-change scores differed according to baseline body weight percentile category, although this association should be interpreted cautiously because of potential regression to the mean, the structurally restricted range of change in the extreme categories, and the limitations of percentile-based analysis. Longitudinal monitoring of body weight percentile trajectories may provide clinically useful information complementary to group-level measures. However, body weight percentile alone does not provide a comprehensive assessment of nutritional status, and additional anthropometric and clinical parameters should be considered when evaluating nutritional status in children undergoing anticancer treatment.
